# MS4A6A is a new prognostic biomarker produced by macrophages in glioma patients

**DOI:** 10.3389/fimmu.2022.865020

**Published:** 2022-08-09

**Authors:** Chunyu Zhang, Haitao Liu, Yinqiu Tan, Yang Xu, Yuntao Li, Shiao Tong, Sheng Qiu, Qianxue Chen, Zhongzhou Su, Daofeng Tian, Wei Zhou, Chunlong Zhong

**Affiliations:** ^1^ Department of Neurosurgery, Shanghai East Hospital, School of Medicine, Tongji University, Shanghai, China; ^2^ Department of Neurosurgery, Huzhou Central Hospital, Affiliated Central Hospital Huzhou Normal University, Huzhou, China; ^3^ Department of Cardiothoracic Surgery, Jiaxing University, The First Affiliated Hospital, Jiaxing, China; ^4^ Department of Neurosurgery, Union Hospital, Tongji Medical College, Huazhong University of Science and Technology, Wuhan, China; ^5^ Department of Neurosurgery, Wuhan University, Renmin Hospital, Wuhan, China; ^6^ Department of Anesthesia, Huzhou Central Hospital, Affiliated Central Hospital Huzhou Normal University, Huzhou, China

**Keywords:** glioma, immune microenvironment, prognosis, immunotherapy, indicator

## Abstract

MS4A6A has been recognized as being associated with aging and the onset of neurodegenerative disease. However, the mechanisms of MS4A6A in glioma biology and prognosis are ill-defined. Here, we show that MS4A6A is upregulated in glioma tissues, resulting in unfavorable clinical outcomes and poor responses to adjuvant chemotherapy. Multivariate Cox regression analysis suggested that MS4A6A expression can act as a strong and independent predictor for glioma outcomes (CGGA1: HR: 1.765, *p* < 0.001; CGGA2: HR: 2.626, *p* < 0.001; TCGA: HR: 1.415, *p* < 0.001; Rembrandt: HR: 1.809, *p* < 0.001; Gravendeel: HR: 1.613, *p* < 0.001). A protein–protein interaction (PPI) network revealed that MS4A6A might be coexpressed with CD68, CD163, and macrophage-specific signatures. Enrichment analysis showed the innate immune response and inflammatory response to be markedly enriched in the high MS4A6A expression group. Additionally, single-cell RNA sequencing (scRNA-seq) analysis revealed distinctive expression features for MS4A6A in macrophages in the glioma immune microenvironment (GIME). Immunofluorescence staining confirmed colocalization of CD68/MS4A6A and CD163/MS4A6A in macrophages. Correlation analysis revealed that MS4A6A expression is positively related to the tumor mutation burden (TMB) of glioma, displaying the high potential of applying MS4A6A to evaluate responsiveness to immunotherapy. Altogether, our research indicates that MS4A6A upregulation may be used as a promising and effective indicator for adjuvant therapy and prognosis assessment.

## Introduction

Glioma is the most common category of cancer in the central nervous system (CNS), with great aggressiveness and neurological destructiveness (1). According to the latest 2021 glioma classification, glioma malignancy is categorized as WHO II–IV based on histological and molecular features (2). Despite the advanced combination of therapeutic regimens and strategies, including surgical resection, radiation treatment, and temozolomide application, outcomes for glioma are still disappointing, especially for glioblastoma (GBM), for which the overall median survival time (MST) is no more than 20 months (3). Therefore, the main objective of the present study was to explore new possible and effective targets for personalized therapeutic management and treatment of glioma.

Publicly accessible data from TCGA, CCGA, and GEO allow for researching clinicopathological features using large-scale tumor samples, which greatly contributes to the detection and identification of effective tumor-associated candidates (4–6). In addition, the widespread application of single-cell RNA sequencing (scRNA-seq) technology has led to the development and establishment of accurate tools with high sensitivity and invasiveness for use in early disease recognition, diagnosis, and treatment. For example, based on scRNA-seq analysis, PDIA5 (Protein Disulfide Isomerase Family A Member 5) has been identified as associated with worse glioma outcomes and induction of macrophage infiltration (7). Recent studies have demonstrated a potential relationship between MS4A6A alterations and aging-related diseases, such as the SNP rs610932, which is located in the 3’ untranslated region of MS4A6A and correlates with cortical and hippocampal atrophy (8). Furthermore, elevated MS4A6A expression in late-onset Alzheimer’s disease (AD) tissues has been identified as correlating with an elevated Braak Tangle Score, a neuropathological measure for AD development and progression (9–11). Recent research has also revealed that MS4A6A dysregulation is involved in the acute phase of Kawasaki disease (KD) *via* macrophage infiltration induction (12). Tumor-associated macrophages (TAMs) are the most representative cell population of tumor-infiltrating leukocytes (TILs) in the glioma immune microenvironment (GIME). TAMs are heterogeneous populations that include brain-resident microglia, border-associated macrophages (BAMs), and bone marrow-derived macrophages (BMDMs), which correlate negatively with infiltration of T cells, neutrophils, and plasmacytoid dendritic cells (pDCs), leading to the immunosuppressive nature of the GIME (13–15). Moreover, intratumoral TAM accumulation is increased along with higher pathological grade in glioma, indicating the critical role of TAMs in tumor development (16, 17). In previous research, *t*-test analysis of 33 subjects showed that dysregulated methylation of MS4A6A may contribute to poor prognosis in GBM; however, the study failed to illustrate the association of methylation and expression of the potential marker (18). Another study on 154 samples proposed that MS4A6A overexpression has no significant correlation with GBM outcomes (*p* = 0.83) (19). As comprehensive large-scale analysis of the biological role of the potential marker MS4A6A in glioma tumorigenesis and prognosis has not been fully performed, there is a lack of in-depth insight.

Here, we report that MS4A6A is hypomethylated and overexpressed in glioma tissue at both transcriptional and protein levels, which is related to a significant decrease in overall survival (OS). We also identified that MS4A6A may promote the level of macrophage infiltration in the GIME.

## Materials and methods

### MS4A6A expression and methylation analysis

TCGA-LGG and TCGA-GBM datasets were obtained, including mRNA expression data, somatic mutation data, and follow-up information from the database TCGA (https://portal.gdc.cancer.gov/). Both sets were merged into a TCGA glioma set for further analysis. Additionally, MS4A6A expression data in the CGGA1 and CGGA2 datasets were acquired from CGGA. The RNA-seq data of TCGA and CGGA glioma datasets were log2(fragments per kilobase of exon model per million fragments mapped (FPKM)+ 1) transformed. MS4A6A microarray expression data in the Gravendeel and Rembrandt datasets were acquired from GlioVis. Clinical data such as age, sex, isocitrate dehydrogenase (IDH) status, 1p19q status, WHO grade, and O⁶-methylguanine-DNA methyltransferase promoter (MGMTp) status were obtained from TCGA and CGGA; information such as age, sex, and WHO grade in Gravendeel and WHO grade in Rembrandt were also obtained. Samples with no survival data were excluded. Finally, a total of 2,089 cases, including 601 samples from TCGA, 965 from CGGA (CGGA1 cohort, *N* = 656; CGGA2 cohort, *N* = 305), 335 from Rembrandt, and 192 from Gravendeel, were included in our research (**Table S1**). Human Protein Atlas (HPA) was utilized to confirm MS4A6A expression levels in glioma tissues at the protein level. To detect the mechanisms of MS4A6A dysregulation, exploration of the mutation status of MS4A6A was conducted using the cBioPortal database. Three subsequent databases were selected for MS4A6A methylation analysis. First, DiseaseMeth version 2.0 (20) was chosen to evaluate MS4A6A methylation differences between glioma samples and nontumor brain tissues. Moreover, we investigated the correlation between the expression and DNA methylation status of MS4A6A based on the MEXPRESS database (21). Expression differences in DNA methyltransferases such as DNMT1 (DNA methyltransferase 1), DNMT3A (DNA methyltransferase 3 alpha), and DNMT3B (DNA methyltransferase 3 beta) between subgroups divided by MS4A6A expression were analyzed using the CGGA database. The workflow of this study is depicted in **Figure S1**.

### Functional annotation of differentially expressed genes related to MS4A6A expression

Patients in the CGGA1 set were divided into two subgroups according to the optimal cutoff obtained by the Survminer package based on MS4A6A expression files. First, DEGs between the subgroups (|log2FC| > 1 and FDR < 0.05) were detected using the R package edgeR and then visualized by volcano plots. Then, DEGs were selected for further analysis using the R package clusterProfiler (22) for Gene Ontology (GO), Kyoto Encyclopedia of Genes and Genomes (KEGG), and Gene Set Enrichment Analysis (GSEA). A PPI network related to MS4A6A was constructed by the STRING database (23).

### Inference of immune infiltrates in the GIME

Infiltrating immune cells constitute a high percentage of nontumor cells in the tumor microenvironment and exert significant effects on cancer biology. For quantification of immune infiltrates in tumor samples, the CIBERSORT algorithm (24) was used with RNA-seq data for the CGGA1 cohort, with 1,000 permutations preset. The TIMER database (25) was selected to calculate the correlation between MS4A6A expression and six types of infiltrating immune cells in the GIME. The ESTIMATE database (26) provides calculated immune scores of TCGA data as representative of infiltrative fractions of immune cells in tumor samples. Immune scores of LGG and GBM patients in TCGA cohorts were divided into two groups, separately, in accordance with the optimal cutoffs of MS4A6A expression.

### Human samples

Human tissues were obtained from the Department of Neurosurgery in Renmin Hospital of Wuhan University from July 2017 to July 2020. Frozen (at −80°C) samples, including 9 normal brain samples and 23 glioma samples, were used for real−time quantitative PCR (RT−qPCR) analysis. A total of 124 paraffin-embedded glioma tissues were selected for immunohistochemical staining (IHC) and immunofluorescence staining. Additionally, nine normal paraffin-embedded brain samples were chosen for IHC. Details of the included samples are shown in **Table S2**. The enrolled patients received no treatment before biopsy. Each subject signed written informed consent before enrollment, and our study received approval from the Institutional Ethical Boards of Wuhan University Renmin Hospital.

### Real−time quantitative PCR analysis

RNA extraction was conducted using the PrimeScriptTM RT Reagent Kit with gDNA Eraser (Takara Bio Inc, Japan) in accordance with the manufacturer’s protocols and transcribed into cDNA for further analysis. RT-qPCR was conducted with SYBR Premix Ex Taq (Takara Bio Inc., Japan). The primer sets are provided in **Table S3**. β-Actin was used for normalization.

### Immunohistochemistry

Sections were deparaffinized, hydrated, and subjected to antigen retrieval in 10 mM sodium citrate (pH 6.0). Endogenous peroxidase was blocked with 3% H_2_O_2_ for 30 min. The sections were blocked with 10% normal goat serum and incubated with primary antibodies (Abcam, America) overnight, followed by incubation with a secondary antibody (Servicebio, China). Signals were evaluated by DAB staining (Servicebio, China). We obtained IHC images using an Olympus BX51 microscope (Olympus). Two independent pathologists scored the slides for the percentage of positive cells per mm^2^ using ImageJ software. IHC scores were evaluated as follows: 0 was considered background staining; 1, 2, and 3 were treated as faint, moderate, and strong staining, respectively. IHC expression was scored as 0–1 for low expression and 2–3 for high expression.

### Immunofluorescence staining

Sections were deparaffinized and hydrated, and antigen was retrieved in 10 mM sodium citrate (pH 6.0); the slides were then washed three times with PBS. Diluted primary antibodies against CD68 (BOSTER, China, dilution ratio: 1:400), CD163 (Abcam, America, dilution ratio: 1:500), and MS4A6A (Abcam, America, dilution ratio: 1:200) were incubated at 4°C overnight, followed by a horseradish peroxidase (HRP)-labeled secondary antibody (SeraCare, China, dilution ratio: 1:200) at 37°C for 1 h under dark conditions. DAPI (ANT046, Antgene) was added in the dark for 5 min, and we obtained IF staining images using a fluorescence microscope (Olympus BX51, Japan).

### Single-cell RNA sequencing analysis

We first detected MS4A6A expression features based on the TISCH database (http://tisch.comp-genomics.org). scRNA-seq data acquisition (GSE138794) was carried out using the GEO database, and 10 samples, including both five LGGs and five GBMs, were subjected to in-depth analysis. Samples were combined using the merge function in the Seurat package. Cells with poor quality (<200 genes/cell, <3 cells/gene, >20% mitochondrial genes, and <10% ribosomal genes) were excluded. Hemoglobin genes were removed due to their low expression levels. Finally, 16,158 genes and 19,667 cells in 10 samples were included in downstream analysis. The top 10 components of principal component analysis (PCA) on the normalized data were subjected to UMAP for dimension reduction, and the scRNA-seq data were processed with the R package Seurat. Specific cell markers were obtained for cell category annotation from the CellMarker database (27) and previous findings (13, 14, 28–34).

### Statistical analysis

Examination of data normality was conducted based on the Shapiro–Wilk test. The Wilcoxon test for nonparametric data and *t*-test for parametric data were used for comparisons between two groups. The optimal cutoff was assessed and acquired using the surv_cutpoint function in the R package Survminer to separate objects into two subgroups in the corresponding independent cohort based on MS4A6A expression. Kaplan–Meier (K-M) curves were plotted. The log-rank test was chosen, and *p*-values were evaluated. The independent predictive potential of MSA4A6 expression was assessed based on multivariate Cox regression analysis. The prediction accuracy of MS4S6A expression for 1-, 3-, and 5-year OS was determined using ROC (receiver operating characteristic) curves. Mutation files of TCGA glioma sets were visualized based on the R package maftools. All statistical analyses were performed using R (v4.1.0). All tests were two-sided, and *p* < 0.05 was considered significant.

## Results

### MS4A6A is overexpressed and hypomethylated in glioma

**Figure 1:**
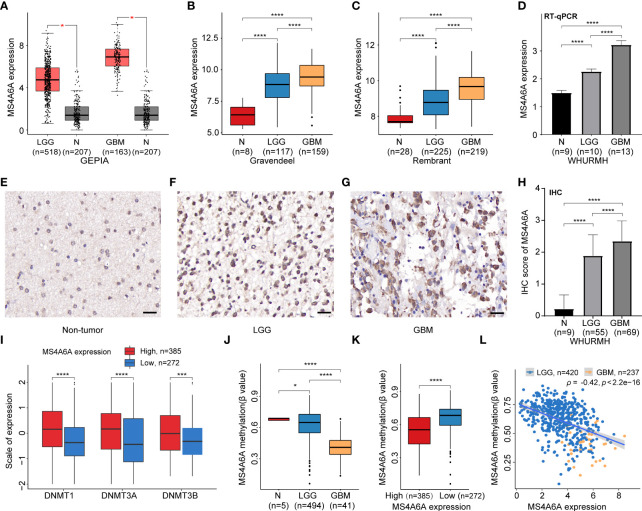
Expression and methylation analysis of MS4A6A in glioma.MS4A6A is upregulated in glioma explored based on public data **(A-C)** and validated by RT-PCR **(D)**; representative IHC staining images of normal control **(E)**, LGG **(F)**, and GBM **(G)**; scale bar, 20 μm. GBM and LGG samples had higher IHC scores than normal samples **(H)**. DNA methyltransferases are upregulated in the high MS4A6A expression group; the expression profiles were Z score normalized **(I)**. The MS4A6A methylation level correlates negatively with glioma WHO grade **(J)** and demonstrated differences between subgroups according to MS4A6A expression **(K)**. MS4A6A methylation has a negative impact on its mRNA expression **(L)**. *p*-values were obtained from the Wilcoxon test **(A–C, I–K)** and *t*-test **(D–H)** (bar plots show means ± SD; ns, *p* > 0.05, *p < 0.05; ***p < 0.001; ****p < 0.0001).

Expression of MS4A6A in TCGA, Rembrandt, and Gravendeel was analyzed and visualized using data from GEPIA and GlioVis. The results showed significant overexpression of MS4A6A in glioma compared with nontumor brain samples; additionally, the elevated level of MS4A6A incrementally correlated positively with glioma WHO grade (**Figures 1A–C).** Furthermore, using the HPA database, we verified that MS4A6A was overexpressed in glioma at the protein level (**Figures S2A–C**). However, we found no significant differences among normal brain, low-grade glioma (LGG), and high-grade glioma (HGG) tissues, which might be due to the small size of the samples included in HPA (**Figure S2D**). RT-qPCR and IHC staining using tissues from healthy controls and LGG and GBM patients validated that MS4A6A is overexpressed in glioma (**Figures 1D–H**).

### Hypomethylation of MS4A6A negatively modulates MS4A6A expression in glioma

**Figure 2:**
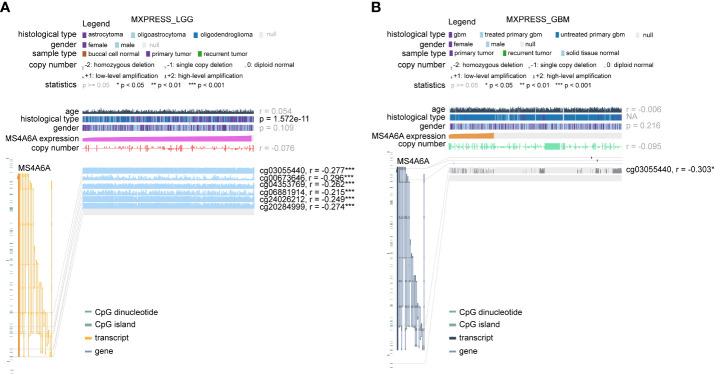
The DNA methylation level of MS4A6A in LGG **(A)** and GBM **(B)** correlates negatively with the MS4A6A expression level using data from the MEXPRESS database. ***P < 0.001.

To further elucidate the aberrant epigenetic and epigenomic mechanisms involved in MS4A6A dysregulation, we first conducted correlation analysis of methylation and expression. Expression levels of DNMT1, DNMT3A, and DNMT3B in the MS4A6A^high^ subgroup were significantly elevated in comparison with those in the MS4A6A^low^ subgroup (**Figure 1I**). Data from the DiseaseMeth database revealed that the degree of MS4A6A methylation was markedly higher in normal brain samples than in gliomas and correlated negatively with pathological grade (**Figure 1J**). In addition, the methylation data for MS4A6A were strongly related to its expression level (**Figure 1K**), which was validated by the Spearman correlation test (ρ = −0.42, *p* < 2.2e−16, **Figure 1L**). Using the MEXPRESS database, we detected six CpG sites (cg20284999, cg24026212, cg06881914, cg04353769, cg00673646, and cg03055440) in the DNA promoter regions of MS4A6A correlating negatively with MS4A6A expression in LGG tissues and that promoter methylation of cg03055440 had a negative effect on MS4A6A expression in GBM tissues (**Figures 2A, B**). Finally, we examined copy number changes in MS4A6A. cBioPortal analysis demonstrated no alterations in MS4A6A (**Figure S2E**). In summary, these findings indicate that MS4A6A is overexpressed in glioma and that hypomethylation is the major epigenetic mechanism leading to overexpression of MS4A6A in glioma.

### Evaluation of prognostic significance and prediction accuracy of MS4A6A

**Figure 3:**
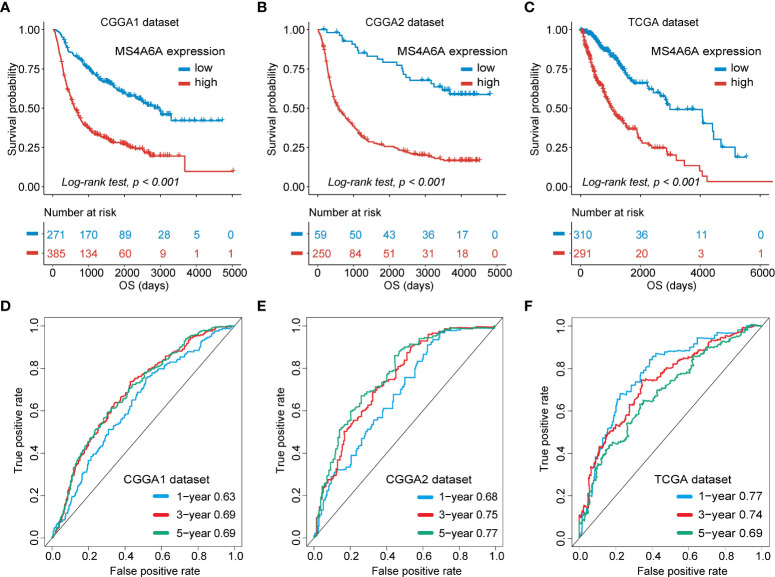
Survival analysis of MS4A6A expression and prediction accuracy assessment.Kaplan–Meier curves of survival differences between MS4A6A subgroups in the CGGA1 set **(A)**, CGGA2 set **(B)**, and TCGA set **(C)**. ROC curves calculating the predictive accuracy of MS4A6A in the CGGA1 set **(D)**, CGGA2 set **(E)**, and TCGA set **(F)** for OS at 1, 3, and 5 years.

To evaluate the prognosis-predicting potential of MS4A6A overexpression, data from TCGA, GlioVis, and CGGA were selected for analysis. According to the optimal cutoff of the expression values of MS4A6A in each dataset, the visualized K-M survival curves and log-rank test confirmed tremendous survival differences between the groups. **Figures 3A–C** and **Figures S3A, B** demonstrate that patients in the low MS4A6A expression group had better outcomes than their counterparts with high MS4A6A expression (log-rank test, *p* < 0.001). Based on AUCs, MS4A6A expression strongly and accurately predicts glioma OS at 1 year (CGGA1: 0.63; GGGA2: 0.68; TCGA: 0.77; Rembrandt: 0.60; Gravendeel: 0.62), at 3 years (CGGA1: 0.69; GGGA2: 0.75; TCGA: 0.74; Rembrandt: 0.65; Gravendeel: 0.69), and at 5 years (CGGA1: 0.69; GGGA2: 0.77; TCGA: 0.69; Rembrandt: 0.68; Gravendeel: 0.68) (**Figures 3D–F** and **Figures S3C, D**). Univariate and multivariate Cox regression analyses of clinicopathological covariates and MS4A6A expression showed MS4A6A to constitute an index that can independently assess glioma outcomes in the CGGA1 (HR: 1.765, *p* < 0.001), CGGA2 (HR: 2.626, *p* < 0.001), TCGA (HR: 1.415, *p* < 0.001), Rembrandt (HR: 1.809, *p* < 0.001), and Gravendeel (HR: 1.613, *p* < 0.001) sets (**Table 1** and **Table S4**). In addition, patients with low MS4A6A had favorable outcomes when they received adjuvant therapy (**Figures S4A–D**).

**Table 1:** Cox regression analysis of the clinical variables, and survival in the CGGA1, CGGA2 and TCGA cohorts.

	CGGA1				CGGA2				TCGA			
	HR	95% CI	*P*		HR	95% CI	*P*		HR	95% CI	*P*		
**Univariate Cox regression analysis**											
Grade	3.791	3.089-4.654	< 0.001		4.888	3.636-6.571	< 0.001		10.884	7.783-15.221	< 0.001		
Gender	1.061	0.868-1.297	0.563		0.924	0.702-1.216	0.572		1.092	0.824-1.447	0.541		
Age	1.026	1.018-1.035	< 0.001		1.033	1.02-1.046	< 0.001		1.074	1.062-1.086	< 0.001		
IDH status	3.093	2.510-3.812	< 0.001		2.777	2.099-3.674	< 0.001		8.840	6.561-11.91	< 0.001		
1p19q status	3.733	2.691-5.177	< 0.001		5.887	3.608-9.606	< 0.001		3.820	2.449-5.957	< 0.001		
MGMTp status	1.257	1.01-1.566	0.041		1.196	0.909-1.573	0.202		3.223	2.419-4.294	< 0.001		
MS4A6A	2.409	1.938-2.993	< 0.001		4.168	2.668-6.511	< 0.001		3.314	2.449-4.484	< 0.001		
**Multivariate Cox regression analysis**											
Grade	2.152	1.594-2.905	< 0.001		2.529	1.799-3.554	< 0.001		2.257	1.506-3.383	< 0.001		
Age	1.009	1-1.018	0.043		1.014	1.001-1.026	0.035		1.052	1.039-1.066	< 0.001		
IDH status	1.472	1.09-1.987	0.012		1.013	0.704-1.459	0.943		2.645	1.667-4.196	< 0.001		
1p19q status	2.021	1.337-3.056	0.001		3.573	2.096-6.09	< 0.001		2.140	1.286-3.562	0.003		
MGMTp status	1.105	0.870-1.404	0.412		1.101	0.820-1.479	0.522		1.187	0.838-1.682	0.334		
MS4A6A	1.618	1.206-2.17	0.001		2.634	1.627-4.264	< 0.001		1.415	1.000-2.004	0.041		

### Association of MS4A6A expression with clinical subgroup

**Figure 4:**
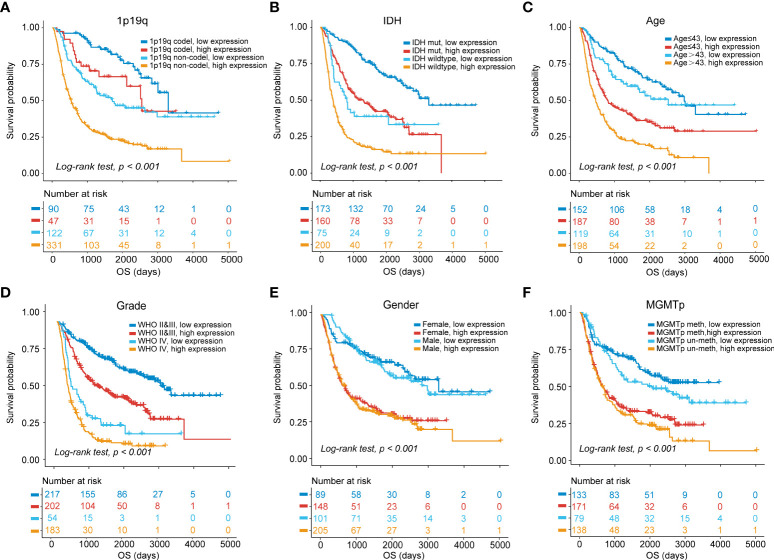
OS stratification analysis.Kaplan–Meier curves of gliomas in the CGGA1 cohort based on the combined effects of MS4A6A expression and 1p19q status **(A)**, IDH status **(B)**, age **(C)**, grade **(D)**, sex **(E)**, and MGMTp status **(F)**.

The prognostic ability of MS4A6A expression was further evaluated in gliomas with distinct clinical and pathological parameters in CGGA1, CGGA2, and TCGA cohorts. We found that MS4A6A expression had no correlation with sex or MGMTp status in glioma. However, 1p19q deletion, IDH wild-type or WHO IV correlated significantly with higher MS4A6A expression (**Figures S5A–C**). Elevated expression of MS4A6A was found in glioma patients with a mean age > 43 years (**Figures S5A–C**), demonstrating the tight association of MS4A6A expression with the aging process. Moreover, according to K-M plots, the survival differences were still obvious after grouping by MS4A6A expression and clinicopathologic subgroups (log-rank test, *p* < 0.001; **Figures 4A–F**, **Figures S6A–F** and **Figures S7A–F**), demonstrating that the MS4A6A expression level might play an important role in glioma OS classification.

### Functional enrichment analysis of MS4A6A-related DEGs

**Figure 5:**
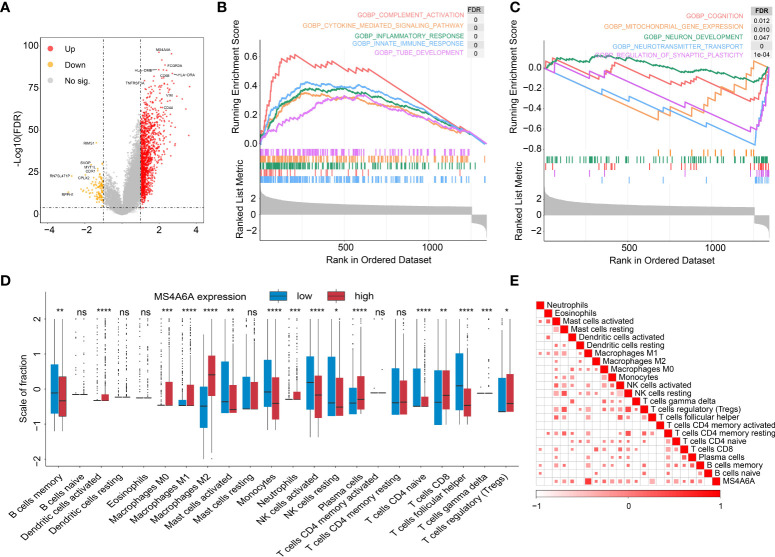
Functional annotation of MS4S6A-related genes and correlation between MS4A6A expression and immune infiltrates.Volcano plot of differentially expressed genes (DEGs) between high and low MS4A6A expression groups **(A)**. GSEA of upregulated **(B)** and downregulated **(C)** DEGs. Box plots of 22 immune cell infiltration levels between the high and low MS4A6A groups **(D)**. Correlation between MS4A6A expression and TILs (tumor-infiltrating lymphocytes) **(E)** (ns, *p* > 0.05, *p < 0.05; **p < 0.01; ***p < 0.001; ****p < 0.0001).

To further explore the potential functions of MS4A6A in glioma, GO and KEGG pathway enrichment analyses were conducted based on DEGs between groups divided by MS4A6A expression. We found that expression of 1,494 genes was dysregulated, including 1,376 upregulated and 118 downregulated genes (**Table S5**). In addition, CD68 (CD68 molecule) and HLA-DRA (major histocompatibility complex, class II, DR alpha) were upregulated in the high MS4A6A expression group (**Figure 5A**). GO analysis of the upregulated genes indicated involvement in immune activation-related processes, such as the MHC protein complex, regulation of mononuclear cell proliferation and antigen processing and presentation, and oncogenic processes, such as extracellular matrix remodeling. KEGG analysis demonstrated that genes with overexpression are mainly enriched in immunosuppressive and carcinogenic pathways, such as the IL-17 and p53 signaling pathways (**Table S6** and **Figure S8A**). Furthermore, GO analysis of downregulated genes showed enrichment of glutamatergic synapse and GABA receptor activity, and KEGG analysis revealed that downregulated genes are associated with cognition (**Table S7** and **Figure S8B**). PPI networks are composed of proteins interacting with each other, and we used the STRING online tool to illustrate potential proteins related to MS4A6A. We found that MS4A6A might be coexpressed with ABCA7 (ATP Binding Cassette Subfamily A Member 7), BIN1 (Bridging Integrator 1), C1orf162 (Chromosome 1 Open Reading Frame 162), CD163 (CD163 Molecule), CD2AP (CD2-Associated Protein), CD33 (CD33 Molecule), FGL2 (Fibrinogen Like 2), MS4A4E (Membrane Spanning 4-Domains A4E), and PICALM (Phosphatidylinositol Binding Clathrin Assembly Protein) (**Figure S8C**). CD163, a phenotypic marker of M2 macrophages, has been applied to differentiate M2 from M1 macrophages, and interactions between CD163 and MS4A6A might indicate the role of MS4A6A in inducing macrophage infiltration. Finally, GSEA was conducted for glioma samples, and GOBP: innate immune response and GOBP: Toll-like signaling pathway were enriched in the MS4A6A high expression group (**Figures 5B, C**). For an in-depth understanding of enrichment differences between MS4A6A subgroups of gliomas in the same WHO grade group, we conducted enrichment analysis on DEGs between MS4A6A LGG and in GBM subgroups (**Tables S8, S9**). The results showed processes including GO: myeloid leukocyte activation and GO: lymphocyte mediated immunity to be significantly enriched in the MS4A6A high expression subgroup in LGG and GBM, respectively (**Tables S10, S11**). These findings show that MS4A6A elevation in glioma might be involved in modulating immune suppression by inducing myeloid leukocyte infiltration, independent of glioma grade.

### Insight into the role of MS4A6A in immune infiltrates in the GIME

It has been demonstrated that immune cells in the tumor microenvironment play a role in carcinogenesis and cancer development. To estimate the possible correlation of MS4A6A with TILs in the GIME, we first carried out correlation analysis on deconvoluted data from the CIBERSORT website and found a strong correlation between the infiltrative levels of macrophages and MS4A6A (**Figures 5D, E**). Using the calculated data from the ESTIMATE database, patients in the MS4A6A^high^ group had statistically higher immune scores than those in the MS4A6A^low^ subgroup in the cohorts TCGA-GBM and TCGA-LGG (**Figure S8D, E**, Wilcoxon test, *p* < 0.001, respectively). As shown in **Figure S8F**, the MS4A6A expression level based on the TIMER database in GBM was significantly related to infiltration of B cells (ρ = 0.378, *p* = 1.13e-15), CD8+ T cells (ρ = −0.362, *p* = 2.26e-14), CD4+ T cells (ρ = 0.147, *p* = 2.60e-03), macrophages (ρ = 0.319, *p* = 2.37e-11), neutrophils (ρ = 0.371, *p* = 4.04e-15), and dendritic cells (ρ = 0.378, *p* = 1.30e−15); for LGG, the MS4A6A expression level was also associated with infiltration of B cells (ρ = 0.445, *p* = 1.16e-24), CD8+ T cells (ρ = 0.233, *p* = 2.73e-07), CD4+ T cells (ρ = 0.691, *p* = 6.04e-69), macrophages (ρ = 0.741, *p* = 2.03e-83), and neutrophils (ρ = 0.668, *p* = 8.98). After adjustment based on glioma purity, MS4A6A remained notably related to the majority of signatures of immune cells, particularly macrophages (GBM: CD68: ρ = 0.623, *p* < 0.001; LGG: CD68: ρ = 0.805, *p* < 0.001) and M2 macrophages (GBM: CD163: ρ = 0.591, *p* < 0.001; LGG: CD163: ρ = 0.750, *p* < 0.001) (**Table 2**). Additionally, there was a high degree of correlation between MS4A6A and the molecular signatures of exhausted T cells, for example, a strong correlation of MS4A6A expression with HAVCR2 (hepatitis A virus cellular receptor 2) expression (GBM: ρ = 0.660, *p* < 0.001; LGG: ρ = 0.714, *p* < 0.001) (**Table 2**), demonstrating the pivotal role played by MS4A6A in HAVCR2-modulated T-cell exhaustion.

**Table 2:** Correlation analysis between MS4A6A expression and related markers of immune cells using data in TIMER database.

Description	Gene markers	GBM		LGG	
		Cor	*P*	Cor	*P*
B cell	CD19	0.316	***	0.375	**
	CD79A	0.405	***	0.42	***
T cell (general)	CD3D	0.582	***	0.543	***
	CD3E	0.544	***	0.595	***
CD8+ T cell	CD2	0.605	***	0.613	***
	CD8A	0.243	**	0.163	***
	CD8B	0.393	***	0.24	***
Monocyte	CD86	0.752	***	0.738	***
	CSF1R	0.59	***	0.568	***
TAM	CCL2	0.406	***	0.466	***
	CD68	0.623	***	0.805	***
	IL10	0.74	***	0.601	***
M1 Macrophage	NOS2	-0.017	ns	-0.233	***
	IRF5	0.392	***	0.669	***
M2 Macrophage	CD163	0.591	***	0.750	***
	VSIG4	0.737	***	0.651	***
	MS4A4A	0.884	***	0.893	***
Neutrophils	CEACAM8	-0.228	**	0.011	ns
	ITGAM	0.398	***	0.593	ns
	CCR7	0.501	***	0.436	ns
Natural killer cell	KIR2DL1	0.184	*	0.043	ns
	KIR2DL3	0.025	ns	0.226	***
	KIR3DL1	0.066	ns	0.071	ns
	KIR3DL2	0.099	ns	0.232	ns
	KIR3DL3	0.084	ns	-0.022	ns
	KIR2DS4	0.118	ns	0.242	ns
Dendritic cell	CD1C	0.384	***	0.511	***
	THBD	0.356	***	0.352	***
	NRP1	0.208	*	0.387	***
	IL3RA	0.029	ns	0.118	**
	ITGAX	0.151	ns	0.484	***
Th1	TBX21	0.29	***	0.372	***
	STAT4	0.245	**	-0.276	***
	STAT1	0.191	*	0.428	***
	IFNG	0.186	*	0.252	***
	TNF	0.23	**	0.227	***
Th2	GATA3	0.175	*	0.42	***
	STAT6	0.265	**	0.348	***
	STAT5A	0.228	**	0.602	***
	IL13	-0.122	ns	-0.015	ns
Tfh	BCL6	-0.153	ns	0.169	ns
	IL21	0.03	ns	0.085	ns
Th17	IL17A	-0.169	*	-0.005	ns
Treg	FOXP3	0.121	ns	-0.126	ns
	CCR8	0.462	***	0.227	ns
	STAT5B	-0.199	*	0.075	ns
	TGFB1	0.319	***	0.608	***
T cell exhaustion	PDCD1	0.362	***	0.568	***
	CTLA4	0.45	***	0.359	***
	HAVCR2	0.66	***	0.714	***
	GZMB	0.373	***	0.342	***

LGG, low grade glioma; TAM, tumor-associated macrophage; Th, T helper cell; Tfh, Follicular helper T cell; Treg, regulatory T cell; Cor, R value of Spearman's correlation; None, correlation without adjustment; Purity, correlation adjusted by purity. ns, P > 0.05; *P < 0.05; **P < 0.01; ***P < 0.001.

### MS4A6A correlates with macrophage infiltration validated by scRNA-seq

**Figure 6:**
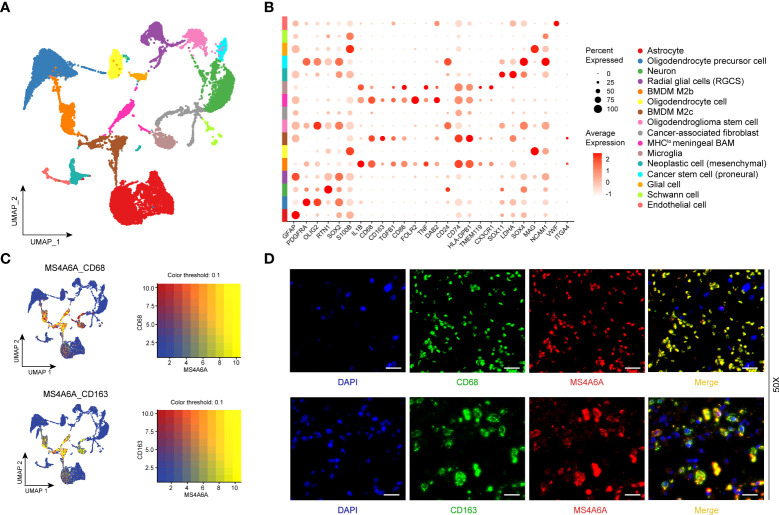
Single-cell RNA-seq analysis and immunofluorescent **(IF)** staining.UMAP plot mapping assigned cell types across glioma samples (*N* = 10). Each cell type is defined by a specific color **(A)**. Dot plot of gene expression of marker genes selected in each subcluster. Rows depict cell types, and columns describe signatures **(B)**. UMAP plots illustrate the coexpression patterns of MS4A6A, CD68, and CD163 **(C)** in scRNA-seq samples. Representative immunofluorescent lf staining images reveal colocalization of MS4A6A/CD68 and MS4A6A/CD163 in glioma tissues. Scale bar, 20 μm **(D)**. Abbreviations: BMDM: bone marrow-derived macrophages; BAM: border-associated macrophages.

To better illustrate the role played by MS4A6A in immune infiltration in the GIME, scRNA-seq analysis was performed. First, based on the six datasets (GSE102130, GSE103224, GSE138794, GSE89567, GSE131928_Smart-seq2, and GSE131928_10X) from the TISCH database, we found MS4A6A to be exclusively expressed in the monocyte/macrophage cluster (**Figures S9A–G**). For further analysis of the main macrophage subpopulations in which MS4A6A is involved, 10 scRNA-seq glioma samples were introduced (**Figure S10A**). A total of 16,158 cells were separated into 16 main clusters by UMAP for nonlinear dimension reduction on the top 10 principal components from PCA (**Figures S10B, C**), with a parameter resolution of 0.10 (**Figure S10D**). **Table S12** demonstrates the profiles of DEGs between each cluster. Sixteen clusters of cells were identified, namely astrocyte, oligodendrocyte precursor cell, neuron, radial glial cell (RGC), bone marrow-derived M2b macrophage (BMDM M2b), oligodendrocyte cell, bone marrow-derived M2c macrophage (BMDM M2c), oligodendroglioma stem cell, cancer-associated fibroblast (CAF), MHC^lo^ meningeal border-associated macrophage (MHC^lo^ meningeal BAM), microglia, neoplastic cell (mesenchymal), cancer stem cell (proneural), glial cell, Schwann cell, and endothelial cell (**Figure 6A**), based on markers retrieved from the CellMarker database and previous findings (13, 14, 28–34). These results confirm that a resolution of 0.10 is biologically valid. The expression profiles of the corresponding markers in all cell clusters are displayed in **Table S13** and visualized in **Figure 6B**. We subsequently analyzed the expression level of MS4A6A in the divided clusters, showing that MS4A6A is mainly expressed in macrophages, including BMDM M2b, BMDM M2c, and MHC^lo^ meningeal BAMs (**Figure 6B**). The three clusters are differentiated cells that have been confirmed to be immunosuppressive cells in the glioma microenvironment (35, 36). We visualized the correlation of MS4A6A/CD68 and MS4A6A/CD163 expression using the blend function of the Seurat R package, which confirmed the expression feature of MS4A6A to be quite dominant in macrophages (**Figure 6C**). Representative IF staining images for CD68/MS4A6A and CD163/MS4A6A confirmed these coexpression and colocalization features in macrophages (**Figure 6D**).

### Correlation between MS4A6A expression and the cancer somatic genome

**Figure 7:**
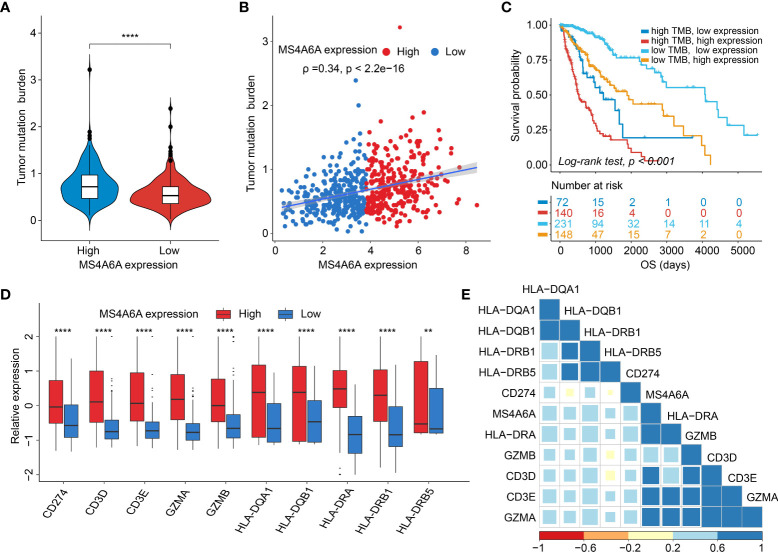
Correlation between the MS4A6A and immunotherapy-related markers.There were TMB differences after grouped by MS4A6A expression **(A)**. Dotplot of correlation of MS4A6A expression with TMB values **(B)**. Kaplan–Meier plot of gliomas OS in the TCGA set stratified by TMB and MS4A6A **(C)**. Box plots of expression features of T cell-inflamed markers between MS4A6A subgroups **(D)**. Corplot of correlation of MS4A6A expression with T cell-inflamed signatures **(E)** (**p < 0.01; ****p < 0.0001).

**Figure 8:**
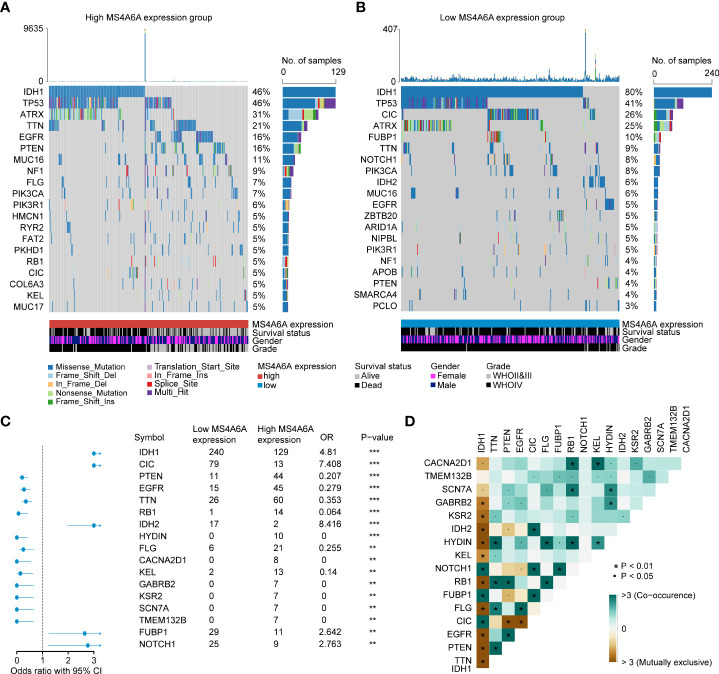
Correlation between the TIMEscore and somatic variants.The oncoPrint plots of gene mutant frequency in high **(A)** and low **(B)** MS4A6A expression group. Forest plot of differentially mutated genes after divided by MS4A6A **(C)**. Corplot of mutually exclusive or co-occurring genes, tested by pairwise Fisher’s exact test **(D)** (*p < 0.05; **p < 0.01; ***p < 0.001).

Recently, many studies have revealed that cancers with an elevated tumor burden mutation (TMB) may show an increased treatment response to anticancer immunotherapeutic strategies (37, 38). Admittedly, MS4A6A expression values and TMB values increase with glioma WHO grade; however, there is no direct research on the relationship of both factors to date. Hence, we made efforts to detect inherent relationships between TMB and MS4A6A expression. As a result, patients with higher MS4A6A expression had significantly increased TMB values that samples with lower MS4A6A expression (Wilcoxon test, *p* < 0.001, **Figure 7A**) and TMB showed a positive correlation with MS4A6A expression (ρ = 0.34, *p* < 2.2e-16, **Figure 7B**). Then, the R package Survminer was selected to acquire the optimal threshold value of TMB to group glioma cases. In terms of the identified interactions of TMB with MS4A6A expression, the synergistic effects of both factors on glioma outcomes were assessed. Based on OS stratification analysis, MS4A6A expression remained an independent prognostic predictor of glioma even when TMB values interfered with (log rank test, *p* < 0.001, **Figure 7C**). These findings demonstrate that MS4A6A might serve as a predictor to select gliomas responsive to antitumor immunotherapy. Further analysis identified the correlation of MS4A6A and immunotherapy-associated signatures, such as immune checkpoint-CD274 (PD-L1), T-cell markers CD3D and CD3E, markers of cytotoxic T lymphocyte (CTL) activation (GZMA and GZMB), and major histocompatibility complex class II (MHC) molecules, such as HLA-DRA and HLA-DRB5 (39–41) (**Figures 7D, E**). The purpose of anticancer immunotherapy is to promote the activity of CTLs within tumors for the development and establishment of an efficient and durable antitumor immune response (42), and recent research demonstrates that PD-L1 expression and MHC II positivity can predict a favorable outcome when PD-1 blockade is applied (40). The positive correlation of the above markers indicates that MS4A6A expression may be an indicator for immunotherapy. In addition, analysis was conducted on the landscape of somatic alterations between the MS4A6A expression groups using the R package maftools. Genes with the top 20 highest variations in the high MS4A6A expression group and low MS4A6A expression group were detected, as displayed in **Figures 8A, B**, respectively. We noticed that TP53 and IDH showed the highest mutation rate in the high MS4A6A expression group (46%, **Figure 8A**), with IDH having the highest mutation frequency in the low MS4A6A expression group (80%, **Figure 8B**). In addition, 17 molecules were differentially altered between the MS4A6A subgroups based on Fisher’s exact test (**Figure 8C** and **Table S14**). Among them, IDH and IDH2 were significantly enriched in the low MS4A6A expression group and PTEN and EGFR in the high MS4A6A expression group (**Figure 8C**). Oncogenic genes are typically symbiotic or demonstrate strong exclusivity in their mutation patterns. We found that IDH1 exhibited a mutually exclusive mutation pattern with that of PTEN and EGFR and concurrent mutant feature with IDH2 mutation, which might help to explain why patients with higher MS4A6A expression had unfavorable outcomes (**Figure 8D**).

## Discussion

In our research, based on comprehensive examination and identification, aging-related MS4A6A is not only able to accurately predict an unfavorable prognosis of glioma but is also associated with malignant features, such as IDH status, 1p19q status, and WHO grade. Additionally, the functions of DEGs were systemically explored, and IL-17 and Toll-like receptor (TLR) signaling pathways were associated with high MS4A6A expression. Additionally, the PPI network demonstrated that CD33, ABCA7, and CD163 might interact with MS4A6A, which may explain why MS4A6A participates in the induction of macrophage infiltration and influences the outcomes of immunotherapy.

Overall, analysis of epigenetic modifications has provided exceptional insight into the tumorigenesis and pathology of brain tumors (43, 44). In methylation analysis, we found lower methylation of MS4A6A in glioma tissues, and this decreased tendency correlated negatively with WHO grade. Importantly, MS4A6A-associated methylation sites (cg20284999, cg24026212, cg06881914, cg04353769, cg00673646, cg03055440, and cg03055440) correlated negatively with expression of the gene. DNA methyltransferases DNMT3B, DNMT3A, and DNMT1 were comparatively overexpressed in the MS4A6A high expression subgroup, which might be the reason for MS4A6A hypomethylation in glioma tissues, leading to insight into upregulated mechanisms of MS4A6A in glioma. Moreover, integrated research of CGGA1 data revealed that MS4A6A expression can independently act as a prognosis-predicting biomarker for glioma, whereby patients with higher MS4A6A expression have poorer outcomes. The above findings were validated in CGGA2, TCGA, Gravendeel, and Rembrandt glioma cohorts. Furthermore, ROC analysis revealed AUC values of approximately 0.70 for 1-, 3-, and 5-year OS, highlighting the strong potential of MS4A6A in clinical assessment of OS. By analyzing interactions of MS4A6A expression with clinicopathological features, we confirmed that the potential marker is markedly downregulated in patients with 1p19q codeletion, IDH mutant status, or WHO grade II/III and upregulated in those aged > 43. These findings show the high possibility of applying MS4A6A expression to stratify glioma clinical and pathological characteristics.

1p19q codeletion has been considered a marker for response to adjuvant chemotherapy and a powerful prognostic predictive marker for LGG (45–47), which might explain why the patients in our research with 1p19q codeletion and lower MS4A6A expression had favorable outcomes after receiving chemotherapy. Regarding IDH mutation, a strong prognosis-predicting and therapeutic response assessment indicator of gliomas (48), our findings revealed a higher mutation frequency in the low MS4A6A expression group, with exclusive mutant features with PTEN and EGFR alterations, which have been identified as crucial changes in glioma genesis and progression (49, 50). Recent research has revealed lower overall levels of TILs in IDH-mutant gliomas than in IDH wild-type gliomas, with decreases in macrophages, T cells, B cells, and dendritic cells (51), consistent with our findings presented in **Figure 5D** and **Figures S5A–C**.

Exploration of the functions of MS4A6A was conducted through GO and KEGG analyses. We identified that MS4A6A might be involved in immune-related biological processes such as antigen processing and presentation and pathways such as the IL-17 signaling pathway and TLR signaling pathway. IL-17 might be involved in anticancer immunosuppression by enhancing the immunosuppressive effect of mesenchymal stem cells (MSCs) (52). IL-17 can also mediate specific γδ T-cell subset recruitment, which promotes immunosuppressive myeloid populations, enhancing cancer progression (53). In addition, IL-17 can promote the proliferation and migration of glioma cells by activating the PI3K/Akt1/NF-κB-p65 pathway (54). Myeloid cells secrete TLRs and produce high levels of immunosuppressive molecules, such as TGF-β, IL-10, and COX-2, to suppress cytotoxic T lymphocyte (CTL) activity (55, 56). The findings above show that MS4A6A overexpression might act as a factor in GIME remodeling, leading to immunosuppression. To gain insight into the immune-related processes of MS4A6A independent of glioma WHO grade, enrichment analysis of MS4A6A in LGG and GBM was conducted separately; in both glioma subgroups, immune-associated processes, such as myeloid leukocyte activation, were enriched in the MS4A6A high expression group. These findings demonstrate that elevated MS4A6A expression might exert a critical role in modulating the antitumor immune response, independent of glioma WHO grade. To explore the protein level of MS4A6A, the PPI network revealed that MS4A6A might interact with the macrophage-related protein CD163 and the glioma progression-related marker fibrinogen-like protein 2 (FGL2). FGL2 upregulation in glioma has been validated as an immune suppressor and is involved in malignant progression (57). CD163 is a classic and distinctive biomarker for macrophage infiltration and is involved in glioma progression and poor survival (58). Furthermore, functional annotation of MS4A6A-related genes using GSEA confirmed the tight correlation of MS4A6A with the innate immune response. Using infiltrative data of immune cells from the TIMER, ESTIMATE, and CIBERSORT algorithms and scRNA-seq analysis, we found that MS4A6A expression is related to macrophage infiltration, including polarized BMDM M2b, BMDM M2c, and MHC^lo^ meningeal BAMs. M2b (Th2 cell activation and immunoregulation) and M2c (immunoregulation, matrix deposition, and tissue remodeling) macrophages are two distinct subsets of alternative macrophage activation (59). Accumulating evidence shows that the tumor microenvironment is complicated and sophisticated, consisting of various cell types roughly divided into malignant and nonmalignant cells and influencing carcinogenesis, tumor growth, and response to clinical interventions (39). A considerable percentage of nonneoplastic cells are TAMs, creating a supporting stromal environment essential for tumor cell growth and invasion (59, 60) by releasing a great variety of chemokines and cytokines, such as TGF-β (transforming growth factor-β), MMP-2 (matrix metalloproteinase-2), and VEGF (vascular endothelial growth factor). Thus, by promoting malignant behaviors, high TAM infiltration results in unfavorable outcomes in glioma, which might be the reason why MS4A6A negatively influences patient prognosis.

Overall, the immune system acts as a modulator of the balance between activation, tolerance, and exhaustion of T cells and tumor pathology by a variety of molecules of coinhibition and costimulation, which are referred to as immune checkpoints (61, 62), such as PD-1 and PD-L1, the dysregulation of which may contribute to evasion of anticancer T-cell immunity (63). Moreover, studies have revealed that blocking this PD-1/PD-L1 signal leads to durable responses and prolonged survival of various tumors (64–66). To gain deep insight into whether stratification based on MS4A6A expression impacts the glioma response to immunotherapy, we first explored and validated the high degree of correlation between MS4A6A and checkpoints at the transcriptome level. Further investigation confirmed a tendency toward a higher TMB in the high MS4A6A expression group. Our findings demonstrate that MS4A6A expression may act as an indicator to assess patients who may benefit from anticancer immunotherapy.

## Conclusion

In summary, great efforts have been made to explore the biology of MS4A6A in glioma. We comprehensively analyzed mechanisms of MS4A6A dysregulation in glioma, highlighting its negative influence on clinical outcomes and further illustrating the differences in macrophage infiltration in association with MS4A6A expression. Our findings show that the features of the inflammatory microenvironment and expression of immune checkpoints might differ based on the MS4A6A expression level and may be relevant to the formulation and conduction of clinical trials to investigate the therapeutic value of MS4A6A in glioma.

## Data availability statement

The original contributions presented in the study are included in the article/**Supplementary Material**. Further inquiries can be directed to the corresponding author.

## Ethics statement

The studies involving human participants were reviewed and approved by the Institutional Ethical Boards of Wuhan University Renmin Hospital (WHURMH). Written informed consent to participate in this study was provided by the participants’ legal guardian/next of kin. Written informed consent was obtained from the individual(s), and minor(s)’ legal guardian/next of kin, for the publication of any potentially identifiable images or data included in this article.

## Author contributions

CZ, LH, YT, YX, YL, QS, ZS and ST performed the data analysis and QC, DT, CZ, and WZ drafted the manuscript. All authors contributed to the article and approved the submitted version.

## Funding

This work was supported by The National Natural Science Foundation of China (No. 81572489) and the Medical Discipline Construction Project of Pudong Health Committee of Shanghai (PWYgy 2021-07) and the Outstanding Leaders Training Program of Pudong Health Bureau of Shanghai (PWR12018-07).

## Conflict of interest

The authors declare that the research was conducted in the absence of any commercial or financial relationships that could be construed as a potential conflict of interest.

## Publisher’s note

All claims expressed in this article are solely those of the authors and do not necessarily represent those of their affiliated organizations, or those of the publisher, the editors and the reviewers. Any product that may be evaluated in this article, or claim that may be made by its manufacturer, is not guaranteed or endorsed by the publisher.
