# Supplementary material for: MS4A6A is a new prognostic biomarker produced by macrophages in glioma patients
Source: Front Immunol. 2022 Aug 9;13:865020. doi: 10.3389/fimmu.2022.865020 (PMC9472524; doi:10.3389/fimmu.2022.865020)
Supplement: Supplementary file 2 [file DataSheet_2.docx]

**MS4A6A is a prognostic biomarker and correlates with macrophage infiltration in glioma**


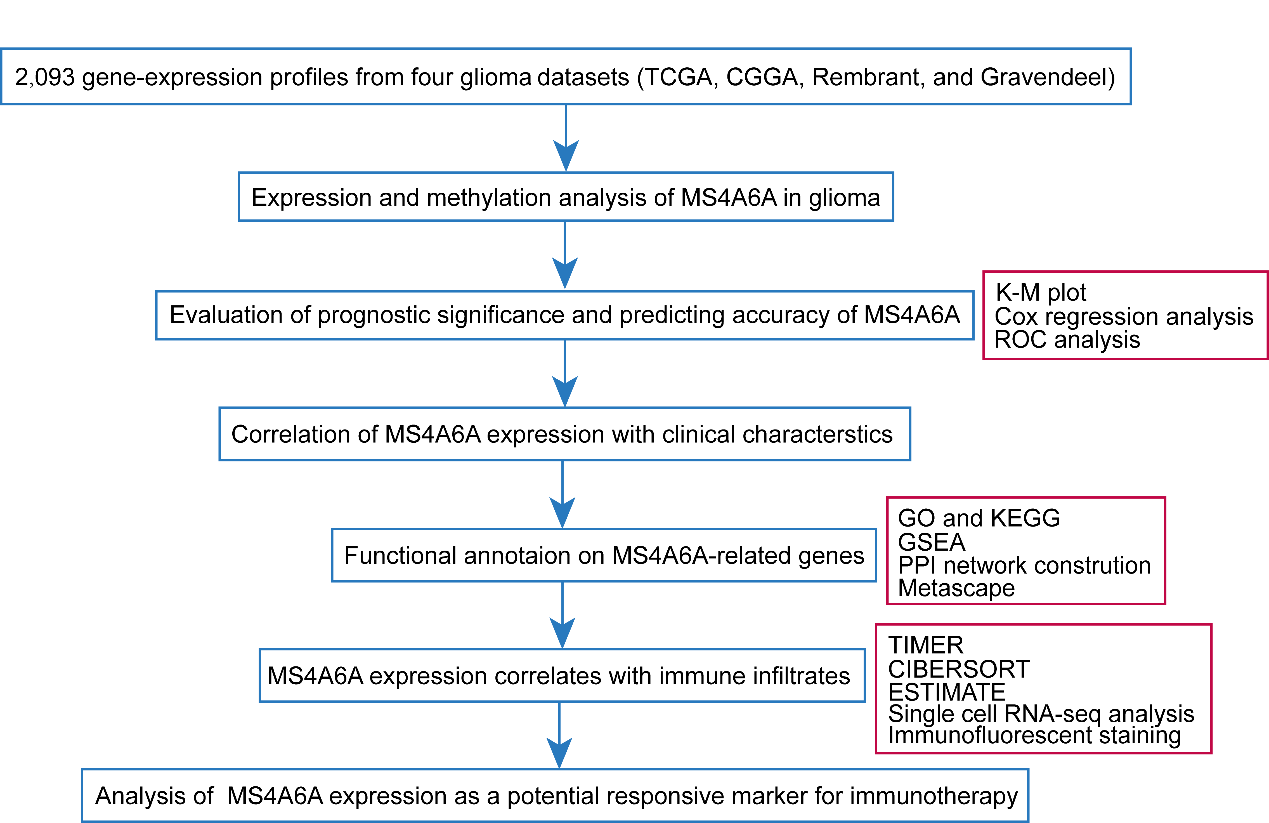


**Figure S1**. Workflow of our research.


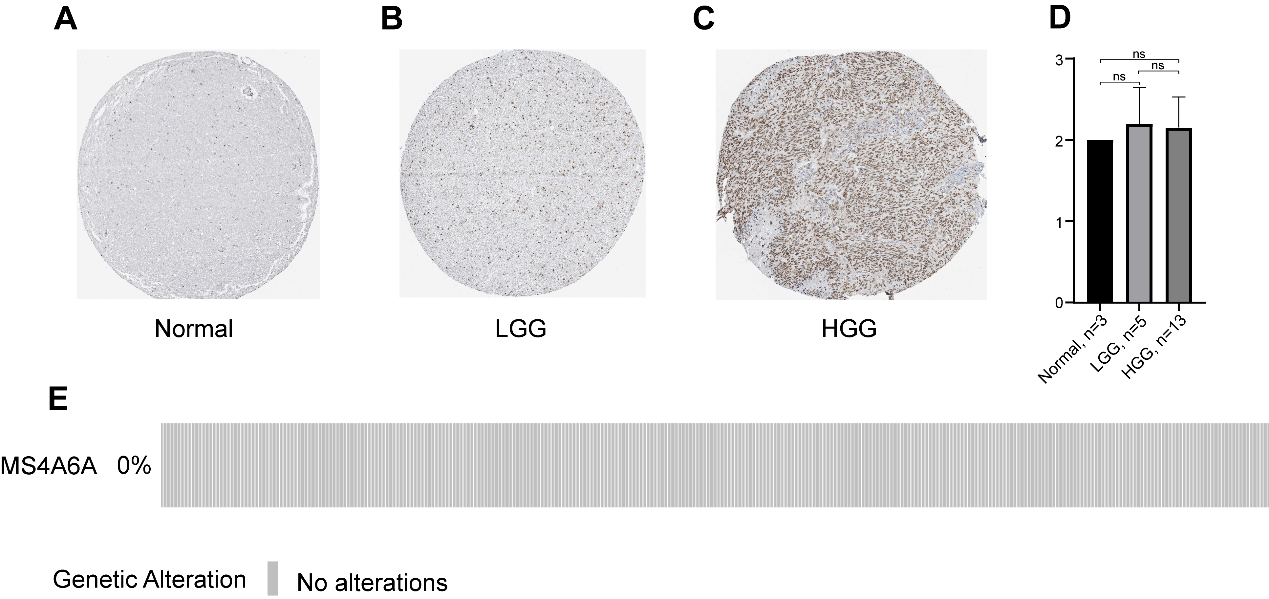


**Figure S2**. MS4A6A protein expression levels in normal brain tissues (A), LGG (B), and GBM (C) based on HPA database and there were no statistical differences among the three groups (D). Oncoprint displayed no genetic alternation of MS4A6A in glioma (E). ns, no significance.


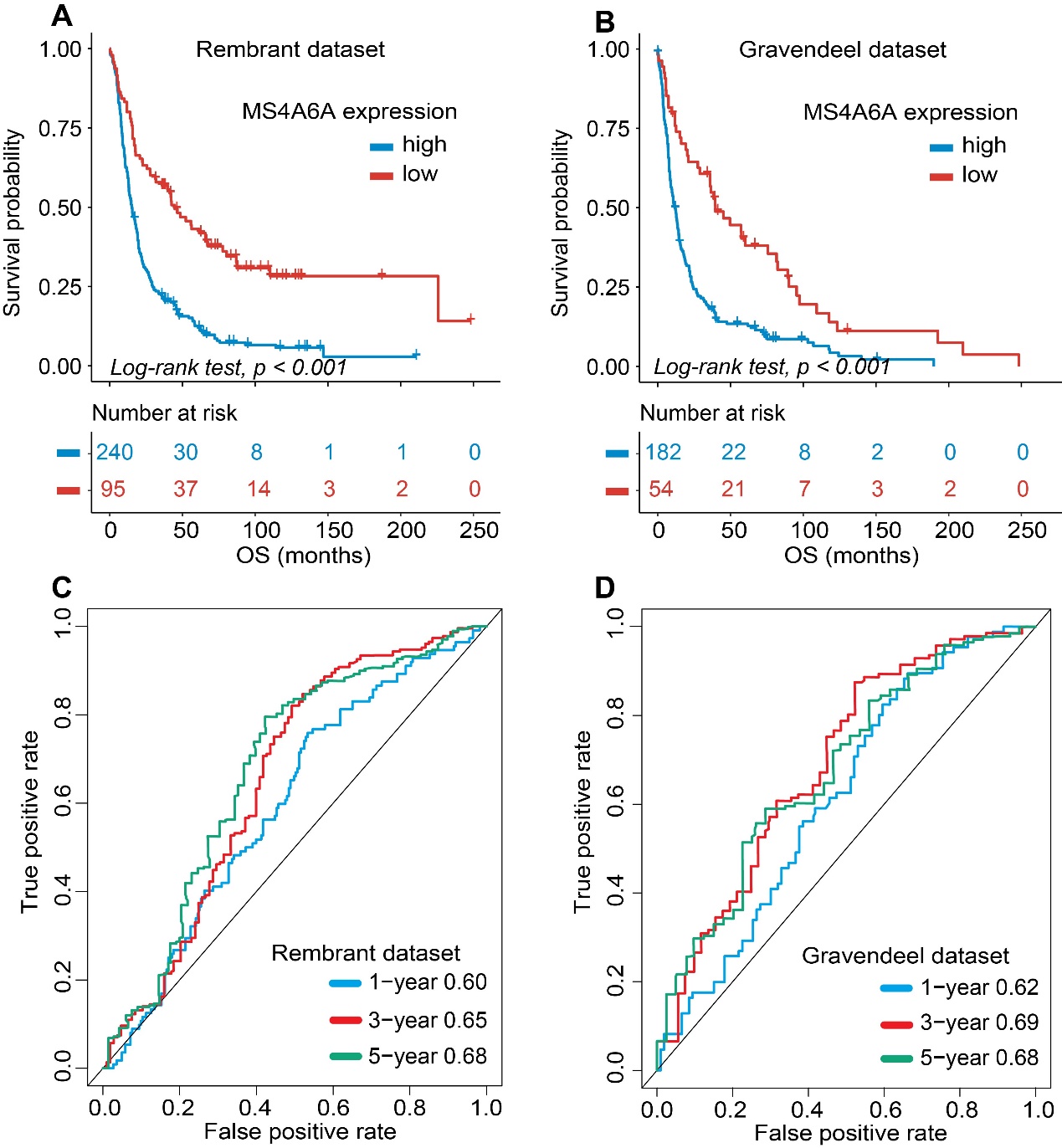


**Figure S3.** Survival analysis of MS4A6A expression and assessment of predicting accuracy

(A-B) Kaplan-Meier curves of survival differences between high and low MS4A6A groups, in Rembrant (A) and Gravendeel (B) sets.

(C-D) ROC curves of estimating the predicting value of the MS4A6A expression in the Rembrant (C) and Gravendeel (D) sets for OS at 1, 3, 5-year.


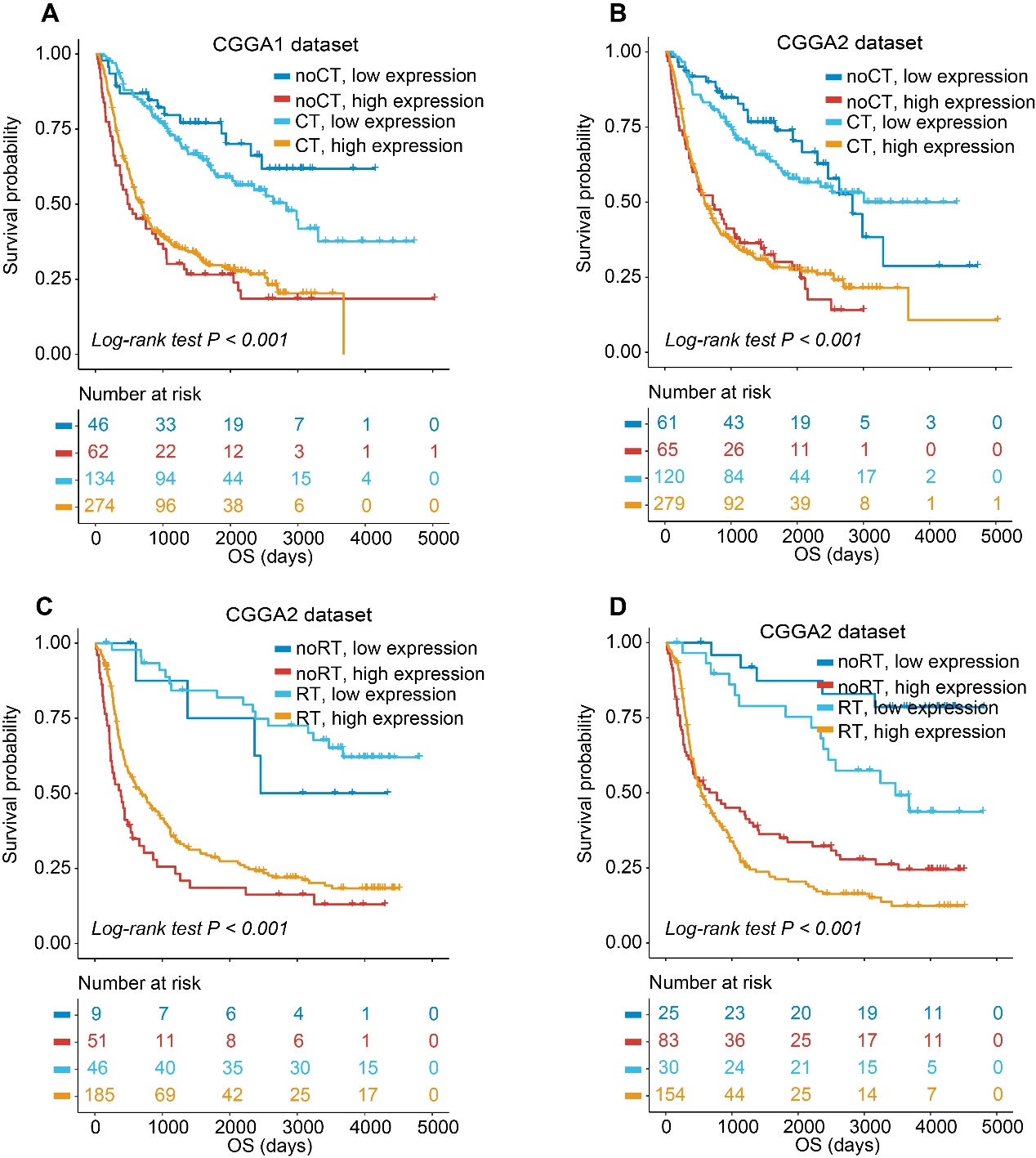


**Figure S4.** Survival analysis of the merged effects of MS4A6A expression and adjuvant therapy.

(A-B) Kaplan-Meier curves of survival differences stratified by MS4A6A expression and adjuvant chemotherapy in CGGA1 and CGGA2 sets.

(C-D) Kaplan-Meier curves of survival differences stratified by MS4A6A expression and adjuvant radiotherapy in CGGA1 and CGGA2 sets.


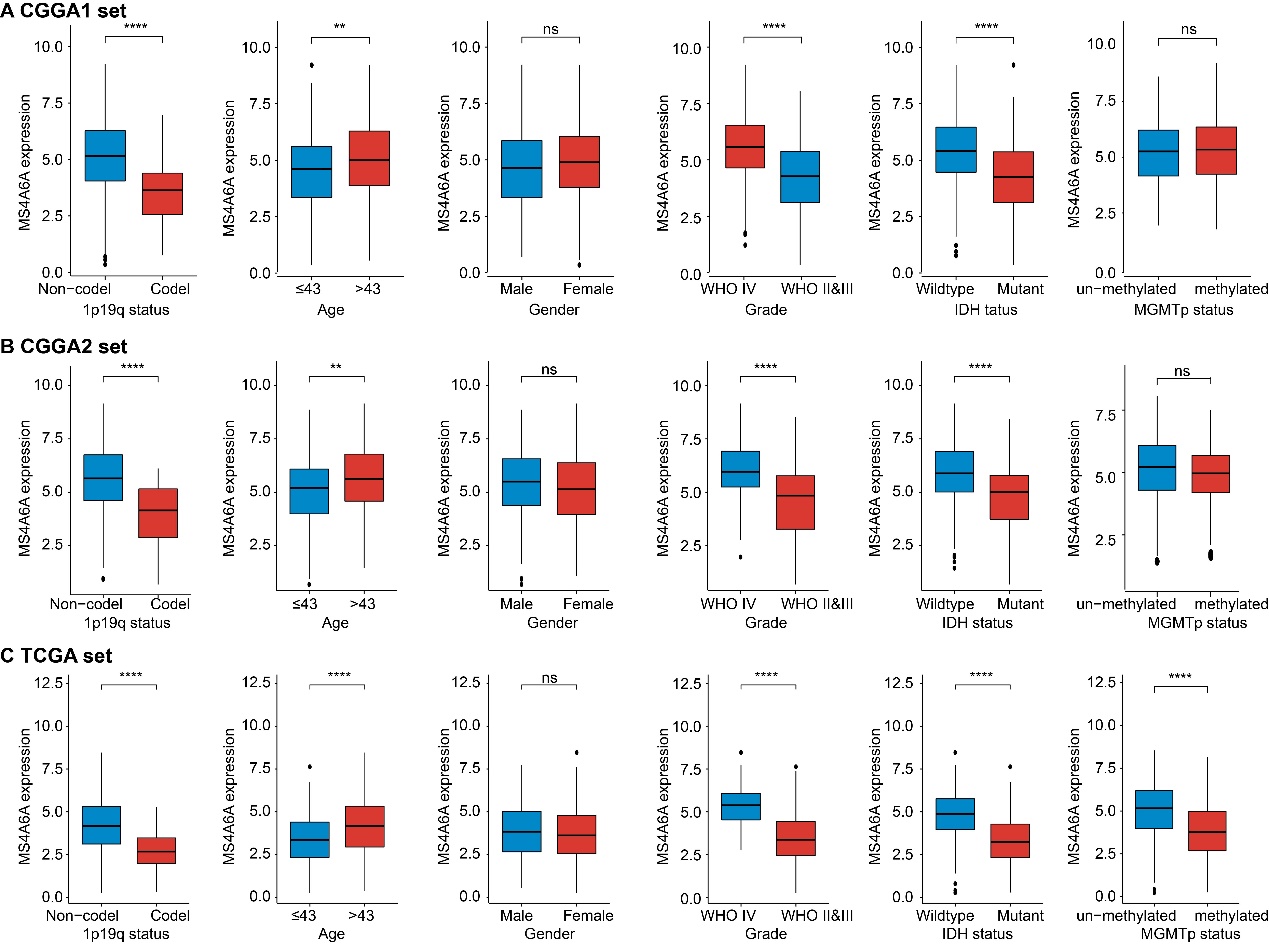


**Figure S5.** Barplots of associations of clinicopathological covariates with MS4A6A subgroups in CGGA1 (A), CGGA2 (B), and TCGA (C) cohorts. (*p < 0.05; **p < 0.01; ***p < 0.001; ****p < 0.0001)


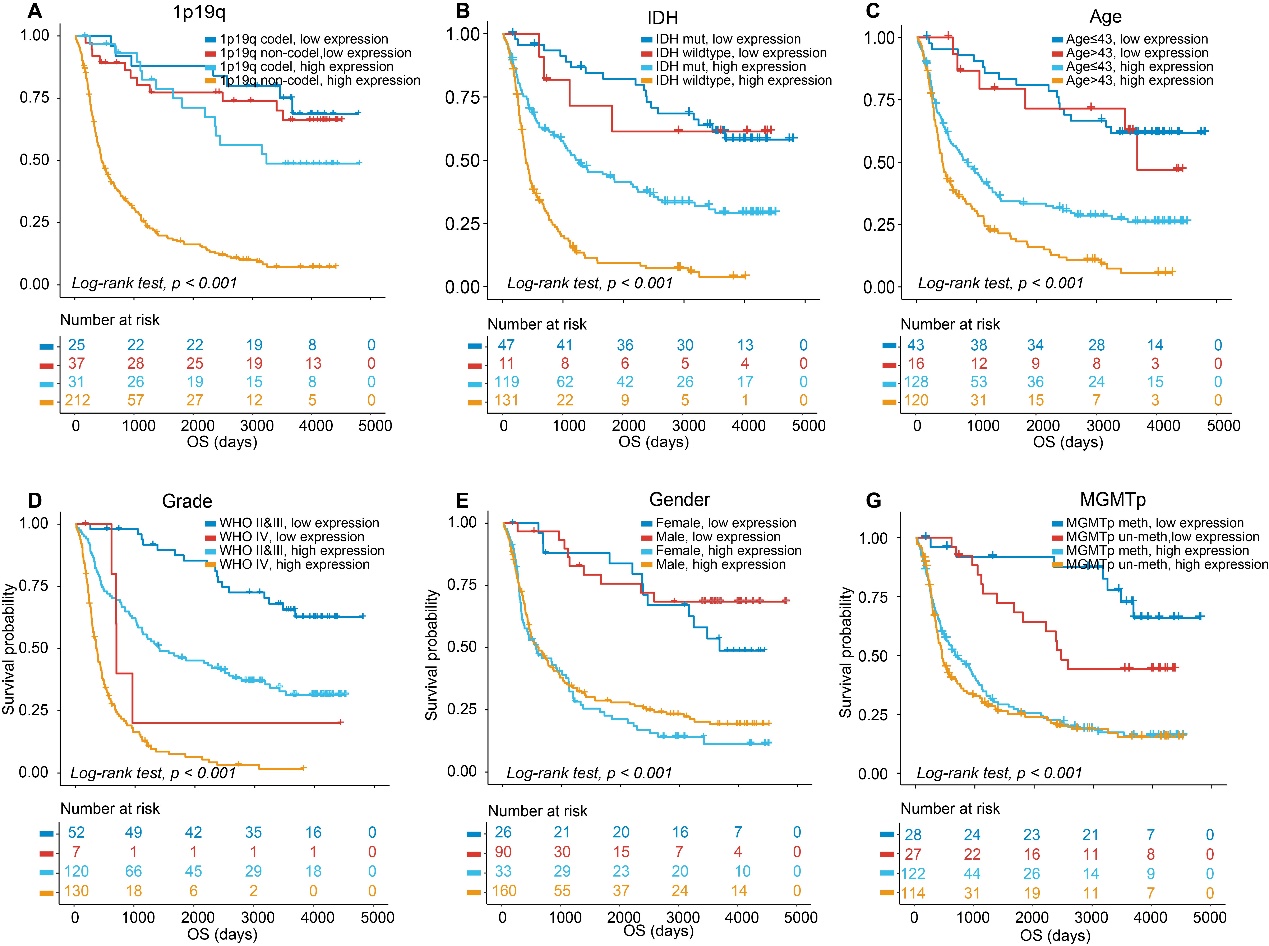


**Figure S6.** (A-F) Kaplan–Meier curves of patients with glioma stratiﬁed by 1p19q status (A), IDH status (B), age (C), grade (D), gender (E), MGMTp status (F) in CGGA2 set.


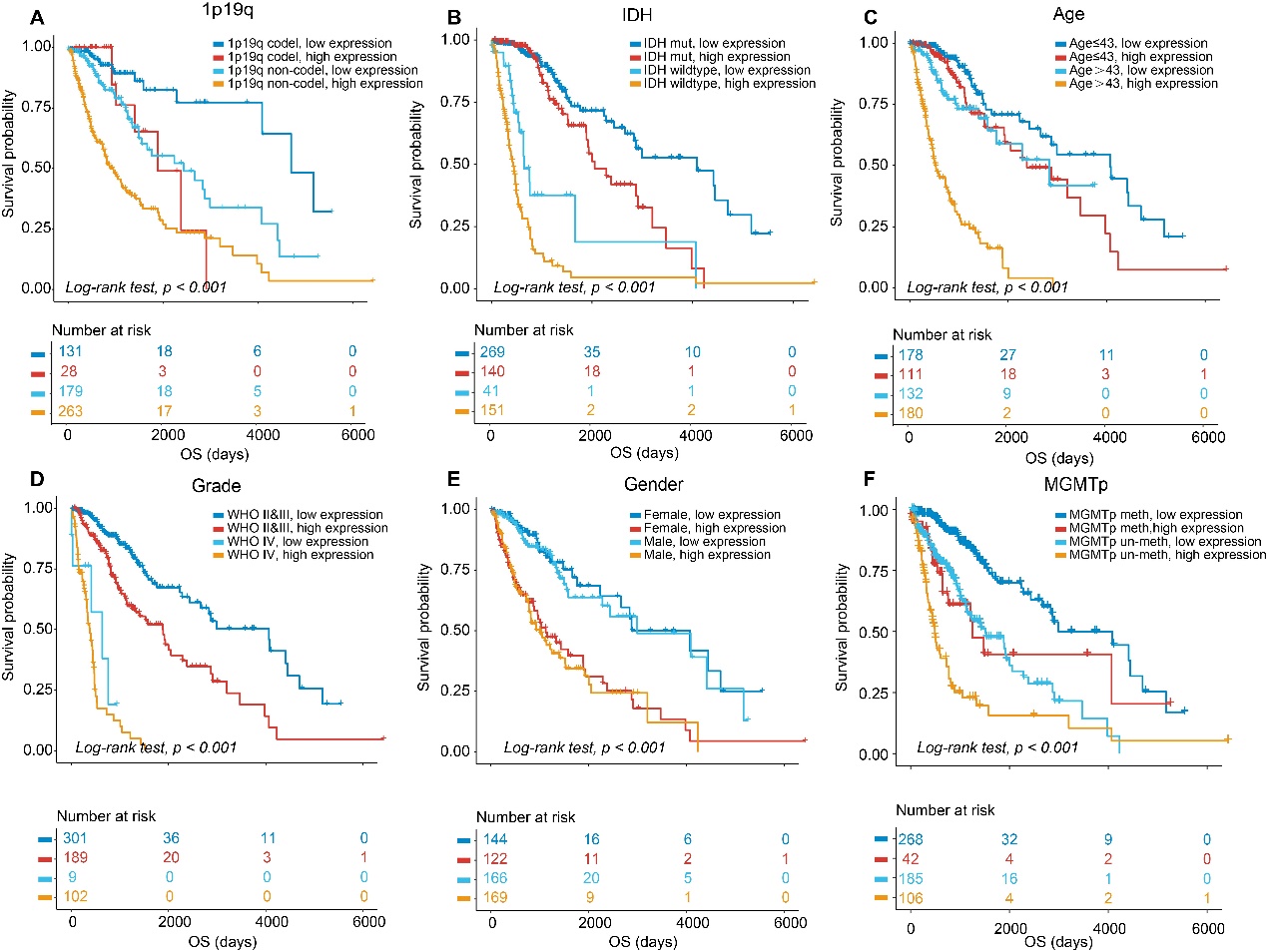


**Figure S7.** (A-F) Kaplan–Meier curves of patients with glioma stratiﬁed by 1p19q status (A), IDH status (B), age (C), grade (D), gender (E), MGMTp status (F) in TCGA set.


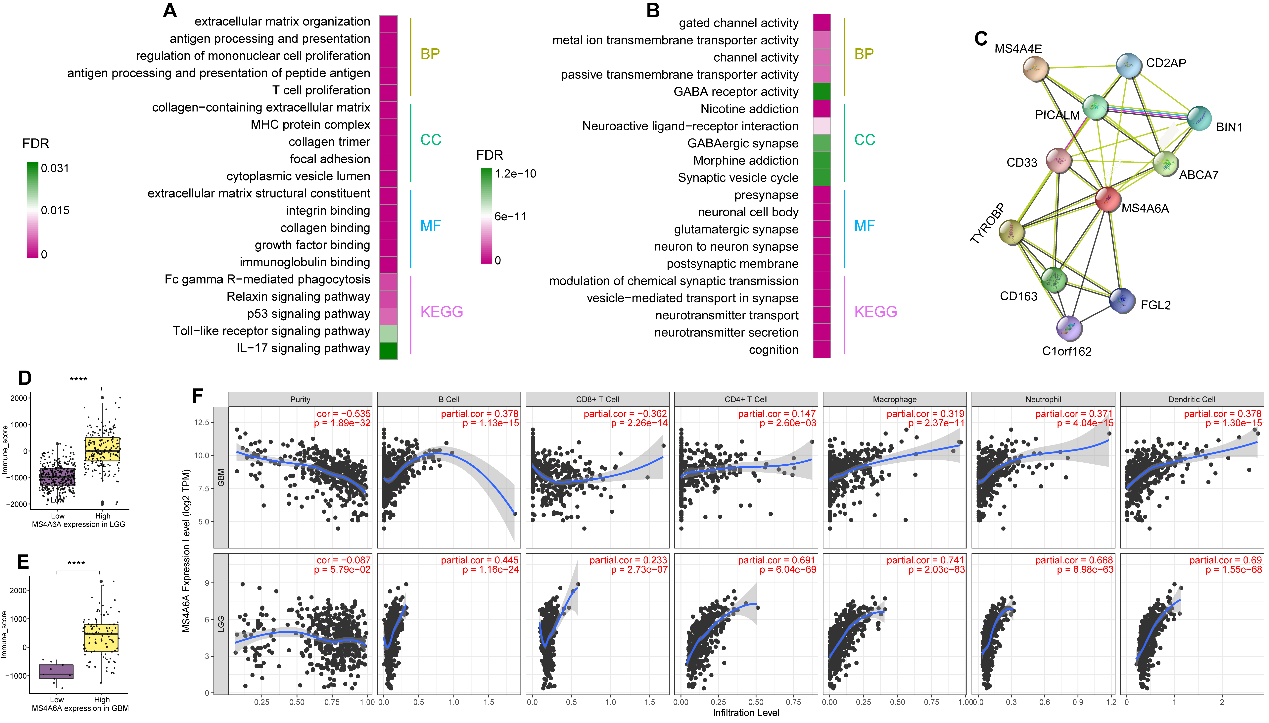


**Figure S8.** Functional annotation of upregulated (A) and downregulated (B) DEGs between the divided MS4A6A groups. Protein-protein network construction of MS4A6A-related genes (C). Barplots of comparison of immune scores between the high and low MS4A6A groups in LGG (D) and GBM (E). Corplots of correlation between MS4A6A expression and six immune infiltrates using TIMER database in LGG and GBM (F). (*p < 0.05; **p < 0.01; ***p < 0.001; ****p < 0.0001)


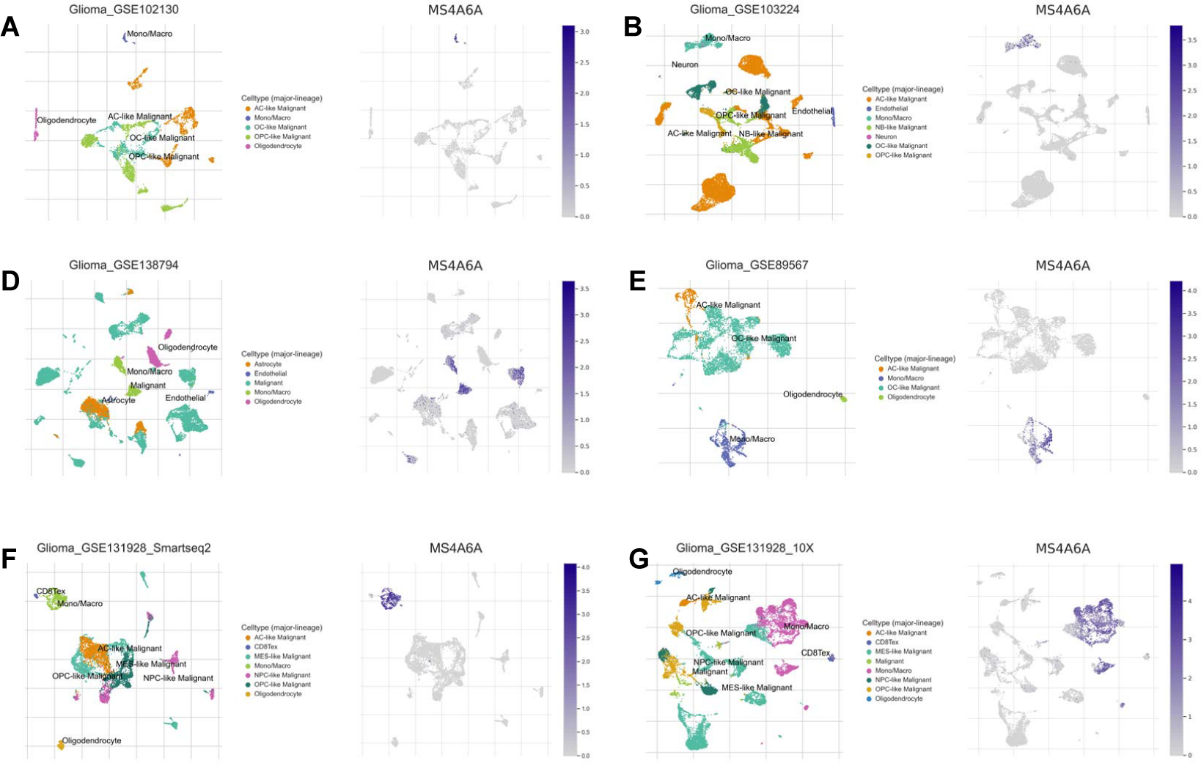


**Figure S9. ScRNA-seq analysis using data from TISCH database.**


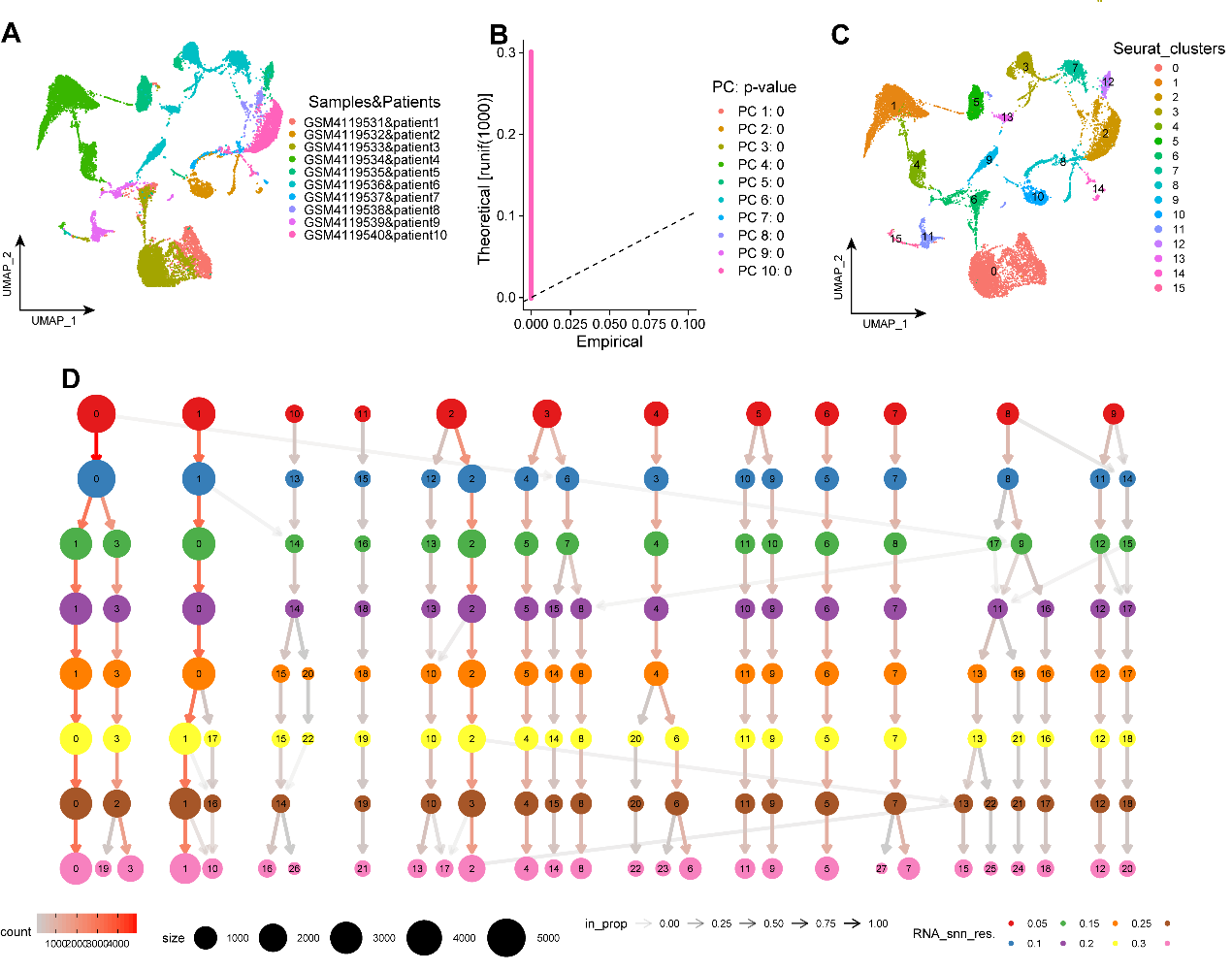


**Figure S10.** UMAP plot of all cells, colored by patients (A). Top ten components in the PCA step, and all were statistically valid (B). UMAP plot mapping multiple subclusters across the single-cell dataset (n = 10), each cluster is defined by a specific color (C). We chose the parameter resolution as 0.10 and there were 16 clusters identified (D).
